# Case report: Undifferentiated sarcoma with multiple tumors involved in Lynch syndrome: Unexpected favorable outcome to sintilimab combined with chemotherapy

**DOI:** 10.3389/fonc.2022.1014859

**Published:** 2022-11-15

**Authors:** Jiaying Liu, Xiaona Chang, Guixiang Xiao, Jingmin Zhong, Bo Huang, Jiwei Zhang, Beibei Gao, Gang Peng, Xiu Nie

**Affiliations:** ^1^ Department of Pathology, Union Hospital, Tongji Medical College, Huazhong University of Science and Technology, Wuhan, China; ^2^ Cancer Center, Union Hospital, Tongji Medical College, Huazhong University of Science and Technology, Wuhan, China

**Keywords:** undifferentiated sarcoma, immune checkpoint inhibitor, sintilimab, lynch syndrome, MSH2, mismatch repair deficiency

## Abstract

**Background:**

Patients with Lynch syndrome are at an increased risk of developing simultaneous or metachronous tumors, while sarcomas have been occasionally reported. Sarcomas are generally not considered part of the common Lynch syndrome tumor spectrum. However, more and more studies and case reports suggested that sarcoma could be a rare clinical manifestation of Lynch syndrome, leading to new treatment strategies for sarcoma.

**Case summary:**

We report the case of a 74-year-old male patient with Lynch syndrome who had rectal mucinous adenocarcinoma and prostate adenocarcinoma and then developed undifferentiated sarcoma of the left neck two years later. Mismatch repair deficiency (dMMR) was confirmed by immunohistochemical staining for the mismatch repair proteins MSH2, MSH6, MLH1 and PMS2. The result of polymerase chain reaction (PCR) microsatellite instability (MSI) testing of sarcoma showed high-level microsatellite instability (MSI-H). Additionally, a pathogenic germline mutation in MSH2 (c.2459-12A>G) was detected by next-generation sequencing (NGS). Taking into account HE morphology, immunohistochemical phenotype, MSI status, NGS result, medical history and germline MSH2 gene mutation, the pathological diagnosis of left neck biopsy tissue was Lynch syndrome related undifferentiated sarcoma with epithelioid morphology. The patient has been receiving immunotherapy (sintilimab) combined with chemotherapy (tegafur, gimeracil and oteracil potassium capsules) and currently has stable disease. We also reviewed the literature to understand the association between sarcoma and Lynch syndrome.

**Conclusion:**

Sarcoma may now be considered a rare clinical manifestation of Lynch syndrome. Attention and awareness about the association between Lynch syndrome and sarcoma need to be increased. Therefore, timely detection of MMR proteins and validation at the gene level for suspicious patients are the keys to avoiding missed or delayed diagnosis and to identifying patients suited for immunotherapy, which may also help to provide appropriate genetic counseling and follow-up management for patients.

## Introduction

Lynch syndrome is a hereditary cancer predisposition syndrome caused by a germline mutation in one of several DNA mismatch repair (MMR) genes (including MLH1, MSH2, MSH6, PMS2) or loss of expression of MSH2 due to deletion in the EPCAM gene (1, 2). Individuals with Lynch syndrome are at an increased risk of developing simultaneous or metachronous tumors, predominantly colorectal cancer and endometrial cancer (3, 4), and are also at increased risk of cancer of the ovary, prostate, stomach, genitourinary system, and hepatobiliary system (2). Moreover, sarcomas are generally not considered part of the common Lynch syndrome tumor spectrum. However, patients with Lynch syndrome have been occasionally reported to develop sarcomas (5–11). As more and more studies and case reports published, the opinion that sarcoma could be a rare clinical manifestation of Lynch syndrome is getting more and more attention, leading to new treatment strategies for sarcoma.

We reported a case in which undifferentiated sarcoma of the neck was identified two years later in a patient with Lynch syndrome who had rectal mucinous adenocarcinoma and prostate adenocarcinoma. The patient has been receiving immunotherapy (sintilimab) combined with chemotherapy (tegafur, gimeracil and oteracil potassium capsules) and currently has stable disease. Furthermore, we also reviewed the literature to understand the association between sarcoma and Lynch syndrome. The report aims to raise awareness of Lynch syndrome-related sarcomas and to identify patients suited for immunotherapy.

## Case presentation

We present the case according to the CARE reporting checklist (**Supplementary Figure S1**; available at https://www.care-statement.org/checklist).

**Figure 1:**
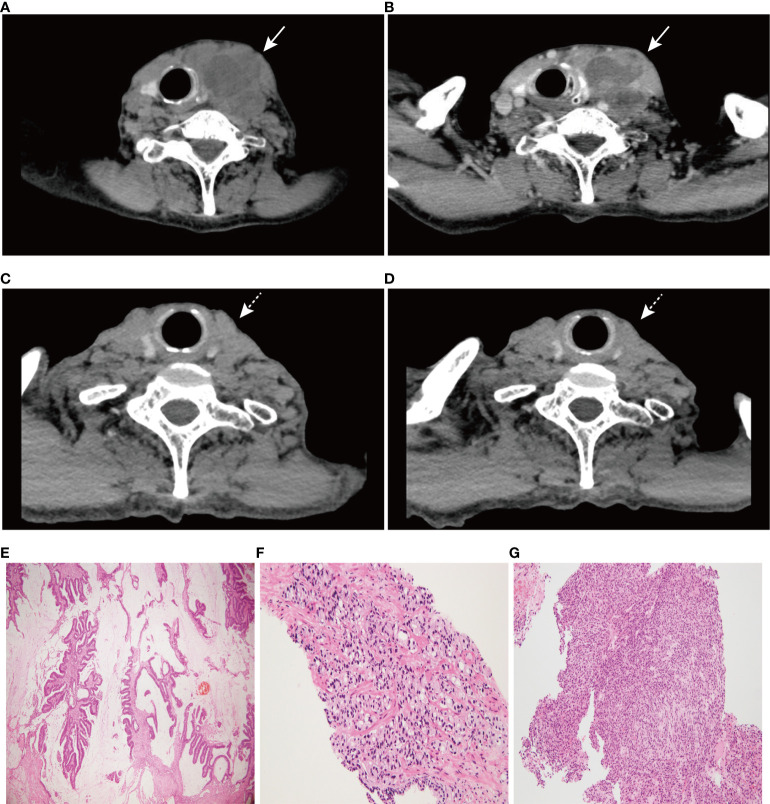
CT scan images of neck and hematoxylin-eosin (HE) staining of three tumors (×100). **(A)** A 66 mm×54 mm round soft tissue mass shadow in the left neck. **(B)** Further contrast-enhanced CT scan showed ring enhancement. **(C)** CT scan image of neck at 6 months post-treatment. **(D)** CT scan image of neck at 10 months post-treatment. **(E)** HE staining of rectal mucinous adenocarcinoma, **(F)** prostate adenocarcinoma, and **(G)** undifferentiated sarcoma of the left neck. Solid arrows indicate tumor masses; dashed arrows indicate significant tumor regression.

A 74-year-old male patient with left neck swelling for one month, without tenderness, and without fever or other symptoms was admitted to Union Hospital affiliated to Tongji Medical College of Huazhong University of Science and Technology in July 2021. A CT-scan of the neck showed a 66 mm×54 mm round soft tissue mass shadow in the left neck (**Figure 1A**), supraclavicular area and superior mediastinum, with multiple enlarged lymph nodes around, and the trachea, thyroid and esophagus were pushed to the right, with local tracheal narrowing. Further contrast-enhanced CT scans showed ring enhancement (**Figure 1B**), and a lack of clear demarcation between the mass and the esophageal wall. In addition, tumor markers were normal.

It is noteworthy that the patient was diagnosed with rectal mucinous adenocarcinoma 30 months ago and subsequently underwent surgery. And there was no special treatment after surgery. Meanwhile, the patient was diagnosed with prostate adenocarcinoma on biopsy (Gleason 3 + 4 = 7) and then received castration therapy. The patient reported no family history of tumors.

**Figure 2:**
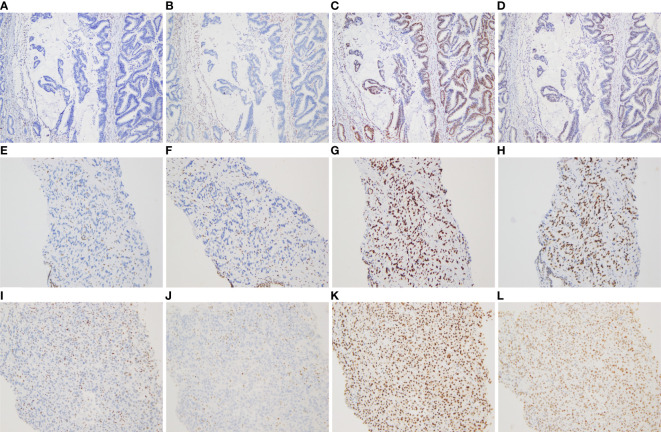
Immunohistochemical staining for DNA mismatch repair proteins (MMR proteins MSH6, MSH2, MLH1, and PMS2) of three tumors (×100). **(A–D)** Absence of MSH6 and MSH2 staining, and positive staining for MLH1 and PMS2 in the rectal mucinous adenocarcinoma; **(E–H)** Absence of MSH6 and MSH2 staining, and positive staining for MLH1 and PMS2 in the prostate adenocarcinoma; **(I–L)** Absence of MSH6 and MSH2 staining, and positive staining for MLH1 and PMS2 in the undifferentiated sarcoma of left neck.

More specifically, the surgical pathology confirmed a diagnosis of rectal mucinous adenocarcinoma (**Figure 1E**), while the tumor invaded through the muscularis propria and into the adipose tissue outside the intestinal wall (pT3), without tumor vascular thrombus or perineural invasion around the tumor, with negative surgical margins. There was no evidence of lymph node involvement (14 lymph nodes were resected), while there was one peri-intestinal cancer nodule. Mismatch repair deficiency (dMMR) was confirmed by immunohistochemical staining for the mismatch repair proteins MSH2, MSH6, MLH1 and PMS2. Immunohistochemistry showed complete loss of MSH2 and MSH6 expression but normal MLH1 and PMS2 expression (**Figure 2**). Germline MSH2 gene mutation (c.2459-12A>G) was detected by next generation sequencing (NGS). NGS result of MSH2 also showed that tumor tissues had higher mutation abundance than the control (84% vs 48%, respectively) (**Supplementary Figure S2**). This genetic variant was located within an intron and did not generally affect the function of protein. It was not represented in the large population databases (1000 Genomes, gnomAD, and ExAC), indicating that this mutation was a rare variant. In addition, the ClinVar database contained six records for the variant, where the pathogenicity was recorded as likely pathogenic of three records and uncertain significance of three remaining records (12–16). In summary, MSH2 gene mutation (c.2459-12A>G) was classified as likely pathogenic according to American College of Medical Genetics and Genomics guidelines (ACMG, 2015). Moreover, gene mutations strongly associated with treatment and prognosis in rectal cancer were also detected by NGS, including KRAS (p. G12D), PIK3CA (p. E545G) and TP53 (p. R248W). Moreover, NGS test of rectal mucinous adenocarcinoma indicated high TMB (68.79 mutations/Mb) and mutations in other mismatch repair related genes (ATM, CDK12, FANCA and MRE11).

The pathological diagnosis of prostate biopsy revealed prostate adenocarcinoma with Gleason 3 + 4 (**Figure 1F**). Similarly, dMMR was also confirmed by immunohistochemical staining. Immunohistochemistry showed complete loss of MSH2 and MSH6 expression but normal MLH1 and PMS2 expression (**Figure 2**). The deficiency of mismatch repair function has several important consequences, such as gain of growth advantage, increase in the point mutation rate, MSI-H and abnormal MMR protein expression by IHC. In summary, we reached consensus that the prostate adenocarcinoma and rectal mucinous adenocarcinoma were associated with Lynch syndrome.

After comprehensive consideration of the patient’s history and status, the patient underwent biopsy of the left neck mass. Microscopically, tumor cells displayed striking atypia and epithelioid morphology, infiltrating into skeletal muscle, without lymph node structure detected (**Figure 1G**). In addition, the lack of differentiation of the immunohistochemical phenotype led to difficulty in understanding the tumor cell of origin. For more details, see **Supplementary Table 1**. Furthermore, NGS, including genes and mutations associated with soft tissue sarcoma typing (57 genes, 236 types of gene fusions and 14 gene mutations), was performed. TP53 mutation (p. Arg175His) was detected, but we were still unable to determine the tumor cell of origin. As expected, dMMR was also confirmed by immunohistochemical staining, consistent with the phenotypes of rectal mucinous adenocarcinoma and prostate adenocarcinoma (**Figure 2**). In addition, the result of polymerase chain reaction (PCR) microsatellite instability (MSI) testing of sarcoma showed high-level microsatellite instability (MSI-H). For more details, see **Supplementary Figure S3**.

In summary, taking into account HE morphology, immunohistochemical phenotype, NGS result, MSI result, medical history and germline MSH2 gene mutation (c.2459-12A>G), the pathological diagnosis of left neck biopsy tissue was Lynch syndrome-related undifferentiated sarcoma with epithelioid morphology.

Among the differential diagnoses, the diagnosis of metastatic cancer of the left neck was ruled out due to a lack of expression of epithelial markers (PCK, CK8/18, CK7, CK20, Villin, CDX2, PSAP, etc.). Lack of expression of malignant melanoma markers (S100, SOX10, HMB45, MelanA, etc.) made it impossible to make the diagnosis of malignant melanoma. Similarly, the absence of detection of lymphatic and hematopoietic system markers (LCA, CD3, CD20, CD38, CD138, MUM1, Kappa, Lambda, MPO, CD43, CD117, etc.) was unable to support the diagnosis of lymphatic and hematopoietic cancer. In addition, the diagnosis of sarcoma with certain differentiation was hard to make due to the absence of lineage-specific markers (Desmin, ERG, CD34, or corresponding fusion genes and mutant genes). In addition, it was unreasonable to make a diagnosis of sporadic undifferentiated sarcoma, which barely demonstrated immunohistochemical absence of MMR proteins and had no pathogenic or likely pathogenic germline gene mutations.

**Figure 3:**
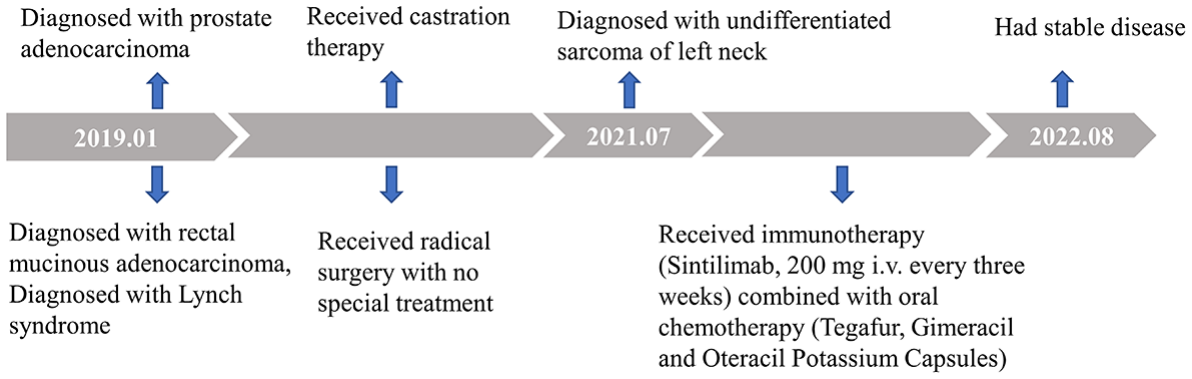
Timeline scheme of the major clinical event of the patient.

The patient has been receiving 15 cycles of immunotherapy (sintilimab, 200 mg i.v. every three weeks) combined with oral chemotherapy (tegafur, gimeracil and oteracil potassium capsules) and well tolerated. Reassuringly, significant regression of the left neck tumor was observed after two cycles of treatment, and the curative effect was evaluated as partial response (PR) according to the RECIST criteria and then maintained the state of PR during a follow-up of 14 months, which further supported our diagnosis (**Figures 1C, D**). The timeline scheme of the major clinical event of the patient is represented in **Figure 3**.

## Discussion

We present a case report of a male patient who was diagnosed with rectal mucinous adenocarcinoma and prostate adenocarcinoma at age 71 and left neck undifferentiated sarcoma at age 74. Immunohistochemical staining for MMR proteins of three tumors yielded consistent results, MSH6 (–), MSH2 (-), MLH1 (+), and PMS2 (+), indicating the presence of dMMR. In addition, the result of PCR MSI testing of sarcoma showed MSI-H. Moreover, the patient carries a germline likely pathogenic MSH2 gene mutation (c.2459-12A>G). All things considered, the final pathological diagnosis of the left neck tumor was Lynch syndrome-related undifferentiated sarcoma with epithelioid morphology.

Sarcoma is a rare clinical manifestation of Lynch syndrome (8, 17, 18). We summarized sarcomas reported in conjunction with Lynch syndrome (except for occasional cases reported in non-English literature) in **Table 1**. A previous study in the Prospective Lynch Syndrome Database showed an increase in the incidence and lifetime risk of sarcoma, although details of specific illness risk and mutated genes were not reported (17). The study enrolled 6,350 patients with Lynch syndrome, and 16 of them developed sarcomas (12 osteosarcomas and 4 soft tissue sarcomas) after 51,646 follow-up years (17), which meant that patients with Lynch syndrome had more than 50-fold and 1.2-fold higher incidence of osteosarcomas and soft tissue sarcomas compared with the expected rates in the general population (osteosarcomas 0.34 per 100,000, soft tissue sarcomas 5.03 per 100,000), respectively (37–39). An Asian study demonstrated tumor development in 55 Japanese Lynch syndrome patients and reported a patient developing sarcoma with germline MLH1 mutation (8). Recently, a cohort study by de Angelis et al. evaluated the occurrence of sarcomas in a cohort of patients with tumors on the Lynch syndrome spectrum and finally identified five eligible cases, three of which carried MSH2 pathogenic variants (18).

**Table 1:** Overview of sarcomas linked to Lynch syndrome published in the literature.

Year	Authors	Sarcoma	Expression of MMR proteins	MSI status	Germline MMR gene mutation
2021	Lam SW et al. (11)	Pleomorphic rhabdomyosarcoma	MSH2 and MSH6 loss	NA	MSH2 p. Cys697Tyr
2020	de Angelis de Carvalho N et al. (18)	Soft-tissue sarcoma	MSH2 and MSH6 loss	NA	MSH2 c.1444A>T; p.Arg482Ter-P
		Osteosarcoma	MSH2 and MSH6 loss	MSI-H	MSH2 c.1661+1G>A-LP
		Myxoid Liposarcoma	Intact	NA	MLH1 exon 17 to 19 deletion-P
		Liposarcoma and Osteosarcoma	MSH2 and MSH6 loss	MSI-H	MSH2 c.2152C>T; p.Gln718Ter-P
2019	Doyle L et al. (19)	Pleomorphic rhabdomyosarcoma	MSH2 and MSH6 loss	NA	MSH2 c.2152C>T; p.Gln718Ter-P
2019	Latham A et al. (20)	Soft-tissue sarcoma	MSH2 and MSH6 loss	MSI-I	MSH2 c.1216C>T; p.Arg406Ter
		Soft-tissue sarcoma	MSH2 and MSH6 loss	MSI-I	MSH2 c.229_230delAG; p.Ser77Cysfs*4
		Soft-tissue sarcoma	NA	MSS	PMS2 del exon 8-9
		Soft-tissue sarcoma	NA	MSS	MSH2 c.942+3A>T
2019	Kazmi S et al. (21)	Malignant phyllodes tumor with stromal or sarcomatous overgrowth	MSH6 partially loss	MSS	MSH6 mutation not specified
2019	Björkman P et al. (22)	Angiosarcoma	MLH1 loss	NA	MLH1 mutation not specified
2018	Tlemsani C et al. (23)	Rhabdomyosarcoma	MLH1 and PMS2 loss	MSS	MLH1 c.1863_1864insT; p.Leu622Serfs*10
2018	Saita C et al. (8)	Sarcoma not specified	Intact	NA	MLH1 mutation not specified
2017	Carnevali IW et al. (24)	Ovary carcinosarcoma	MSH6 loss	MSI-H	MSH6 c.931_935delAAAAG; p.Lys311Glufs*4
2016	Nguyen A et al. (25)	Myxofibrosarcoma	MLH1 and PMS2 loss	MSI-H	MLH1 c.678-7_686del16
2015	Schiavi A et al. (26)	Leiomyosarcoma	MSH2 and MSH6 loss	NA	MSH2 c.649dupA; p.Ile217Asnfs*15
		Leiomyosarcoma	NA	NA	MLH1 c.2195_2198dupAACA
2013	Yozu M et al. (27)	Pleomorphic liposarcoma	MSH2 and MSH6 loss	NA	MSH2 mutation not specified
2012	Urso E et al. (28)	Leiomyosarcoma	MSH2 and MSH6 loss	MSS	MSH2 del exon 1–16
2011	Brieger A et al. (5)	Malignant fibrous histiocytoma	MSH2 loss	MSI-H	MSH2 c.2038C>T; p.Arg680Ter
		Malignant fibrous histiocytoma	MSH2 loss	MSI-H	MSH2 c.942+3A>T
2009	Yu VP et al. (29)	Leiomyosarcoma	MLH1 loss	MSI-H	MLH1 c.200G>A; p.Gly67Glu
2009	Nilbert M et al. (30)	Sarcoma not specified	NA	NA	MSH2 c.145_148delGACG; p.Asp49Argfs*14
		Liposarcoma	MSH2 and MSH6 loss	MSS	MSH2 c.942+3A>T
		Sarcoma not specified	NA	NA	MSH2 c.942+3A>T
		Carcinosarcoma	MSH2/MSH6 loss	MSI-H	MSH2 c.1165C>T; p.Arg389Ter
		Gliosarcoma	MSH2 and MSH6 loss	NA	MSH2 c.1696_1697delAAinsG; p.Asn566Valfs*24
		Liposarcoma	MSH2 and MSH6 loss	NA	MSH2 c.1-?_366+?del
		Chondrosarcoma	Intact	NA	MLH1 c.1204A>T; p.Lys402Ter
		Sarcoma not specified	NA	NA	MLH1 c.1204A>T; p.Lys403Ter
		Osteosarcoma	NA	NA	MLH1 c.1276C>T; p.Gln426Ter
		Liposarcoma	NA	NA	MLH1 c.1732+?_c.2268del
		Carcinosarcoma	MSH6 loss	NA	MSH6 c.1085delC; p.Pro362Leufs*9
		Leiomyosarcoma	NA	NA	MSH6 c.3514_3515insA; p.Arg1172Lysfs*5
		Malignant hemangiopericytoma	Intact	NA	MSH6 c.3850_3851insATTA; p.Thr1284Asnfs*6
2008	Geary J et al. (31)	Soft-tissue sarcoma	MLH1 loss	NA	MLH1 mutation not specified
2007	South SA et al. (32)	Carcinosarcoma	MLH1 loss	NA	MLH1 c.1896G>C; p.Glu632Asp
2006	Hirata K et al. (33)	Liposarcoma	MSH2 loss	NA	MSH2 c.677delAT; p.Arg227Glufs*19
2003	Lynch HT et al. (34)	Osteosarcoma	NA	MSI-H	MSH2 exon 4 splice site mutation
		Malignant fibrous histiocytoma	NA	MSI-H	MSH2 del exon 3–8
2003	den Bakker MA et al. (35)	Rhabdomyosarcoma	MSH2 loss	MSI-H	MSH2 mutation not specified
2000	Sijmons R et al. (36)	Malignant fibrous histiocytoma	MSH2 loss	MSI-H	MSH2 p.Gly429Ter

NA, not available; MSI-H, high microsatellite instability; MSI-I, indeterminate microsatellite instability; MSS, microsatellite stability.

Some previous studies indicated that the development of sarcoma in patients with Lynch syndrome was associated with the expression of MMR proteins, thereby connecting sarcoma with MMR genes (5, 7, 9–11, 28, 33). Furthermore, previous studies have shown that MMR genes may be associated with sarcoma risk (18, 40–42). A study of cancer susceptibility variants based on The Cancer Genome Atlas (TCGA) data described that two MSH2 mutation carriers were detected in an unselected sarcoma population (225 patients) and classified MSH2 as potentially associated with sarcoma risk according to variant burden analysis (odds ratio, 9.9; *p* = 0.02; false discovery rate, 0.09) (40). In addition, Mirabello et al. analyzed pathogenic germline variants in cancer-susceptibility genes in 1244 patients with osteosarcoma and found more germline MSH2 pathogenic variants in patients with osteosarcoma than in the control group (*p* < 0.05) (41). Moreover, a previous study showed that sarcoma tended to be more associated with pathogenic variants of MSH2 than other MMR genes, as 25 of 43 (58.1%) tested cases had MSH2 germline mutations (18). It was a significantly higher frequency in patients with sarcoma than in unselected patients with Lynch syndrome, where MSH2 was usually the second most frequently mutated gene (seen in approximately 40% of patients) (17, 43).

With the advent of the era of tumor immunity, immune checkpoint inhibitor therapy has become an effective treatment for microsatellite instability-high (MSI-H) or dMMR tumors (44, 45). Latham A et al. assessed the MSI status of 15,045 patients (more than 50 cancer types) based on NGS data, and the incidence of MSI-H and MSI-indeterminate (MSI-I) in soft tissue sarcomas was found to be 5.7% (45/785), while two of them were diagnosed with Lynch syndrome with pathogenic MSH2 variants (20). Similarly, another recent study based on NGS data reported that the incidence of dMMR in an unselected cohort of adult soft tissue and bone sarcomas was 2.3% (7/304) (19). Somatic mutation analysis showed that all seven patients had MMR gene mutations (4 of MSH2 or EPCAM, 2 of PMS2, 1 of MSH6), and further germline sequencing of three patients (2 of MSH2, 1 of MSH6) suggested that one patient had pathogenic MSH2 germline mutation and was also diagnosed with Lynch syndrome (19). Tlemsani C et al. highlighted the importance of identifying Lynch syndrome in patients with sarcoma (23). The article described a 19-year-old male patient who presented with metastatic chemoresistant pleomorphic rhabdomyosarcoma. Then, the patient received anti- programmed death (PD)-1 antibody therapy (nivolumab) due to detection of the MLH1 germline pathogenic variant and achieved a rapid complete response of the lung metastases, which appeared sustained after a 1-year follow-up (23). Furthermore, data from the phase II KEYNOTE-158 study of pembrolizumab (an anti-PD-1 monoclonal antibody) in patients with previously treated, advanced noncolorectal MSI-H/dMMR cancer (including 14 sarcomas) demonstrated the clinical benefit of anti-PD-1 therapy among patients with sarcoma (46).

In the present case, undifferentiated sarcoma of the left neck was identified two years later in a 74-year-old male patient with Lynch syndrome who had rectal mucinous adenocarcinoma and prostate adenocarcinoma. The conventional chemotherapy drugs for undifferentiated sarcoma were adriamycin, ifosfamide, gemcitabine, paclitaxel, etc (47–49). The patient refused all intravenous chemotherapy due the older age. Despite the lack of reliable evidence, there were several studies showed that the fluorouracil was effective against undifferentiated sarcoma (50, 51). Therefore, the patient has been receiving immunotherapy (sintilimab) combined with chemotherapy (tegafur, gimeracil and oteracil potassium capsules). Reassuringly, significant regression of the left neck tumor was observed, and the patient was in good condition after a follow-up of 14 months.

In conclusion, sarcoma may now be considered a rare clinical manifestation of Lynch syndrome. Although the risk of sarcoma was significantly lower than that of other common Lynch syndrome-associated tumors, attention to and awareness of the association between Lynch syndrome and sarcoma need to be increased. Therefore, timely detection of MMR proteins by IHC and validation at the gene level for suspicious patients are the keys to avoiding missed or delayed diagnosis and to identifying patients suited for immunotherapy, which may also help to provide appropriate genetic counseling and follow-up management for patients.

## Ethics statement

Ethical review and approval was not required for the study on human participants in accordance with the local legislation and institutional requirements. The patients/participants provided their written informed consent to participate in this study. Written informed consent was obtained from the individual(s) for the publication of any potentially identifiable images or data included in this article.

## Author contributions

Conception/Design: XN and XC; Provision of study material or patients: GP, GX and JinZ; Collection and/or assembly of data: XC and JL; Data analysis and interpretation: JL; Manuscript writing: JL and XC; Final approval of manuscript: GP and XN. All authors have read and approved the submitted version of the manuscript.

## Funding

The research was supported by Union Hospital, Tongji Medical College, Huazhong University of Science and Technology Research Fund. (2021xhyn080).

## Conflict of interest

The authors declare that the research was conducted in the absence of any commercial or financial relationships that could be construed as a potential conflict of interest.

## Publisher’s note

All claims expressed in this article are solely those of the authors and do not necessarily represent those of their affiliated organizations, or those of the publisher, the editors and the reviewers. Any product that may be evaluated in this article, or claim that may be made by its manufacturer, is not guaranteed or endorsed by the publisher.
