# Supplementary figures and images for: Case report: Undifferentiated sarcoma with multiple tumors involved in Lynch syndrome: Unexpected favorable outcome to sintilimab combined with chemotherapy
Source: Front Oncol. 2022 Nov 15;12:1014859. doi: 10.3389/fonc.2022.1014859 (PMC9706001; doi:10.3389/fonc.2022.1014859)

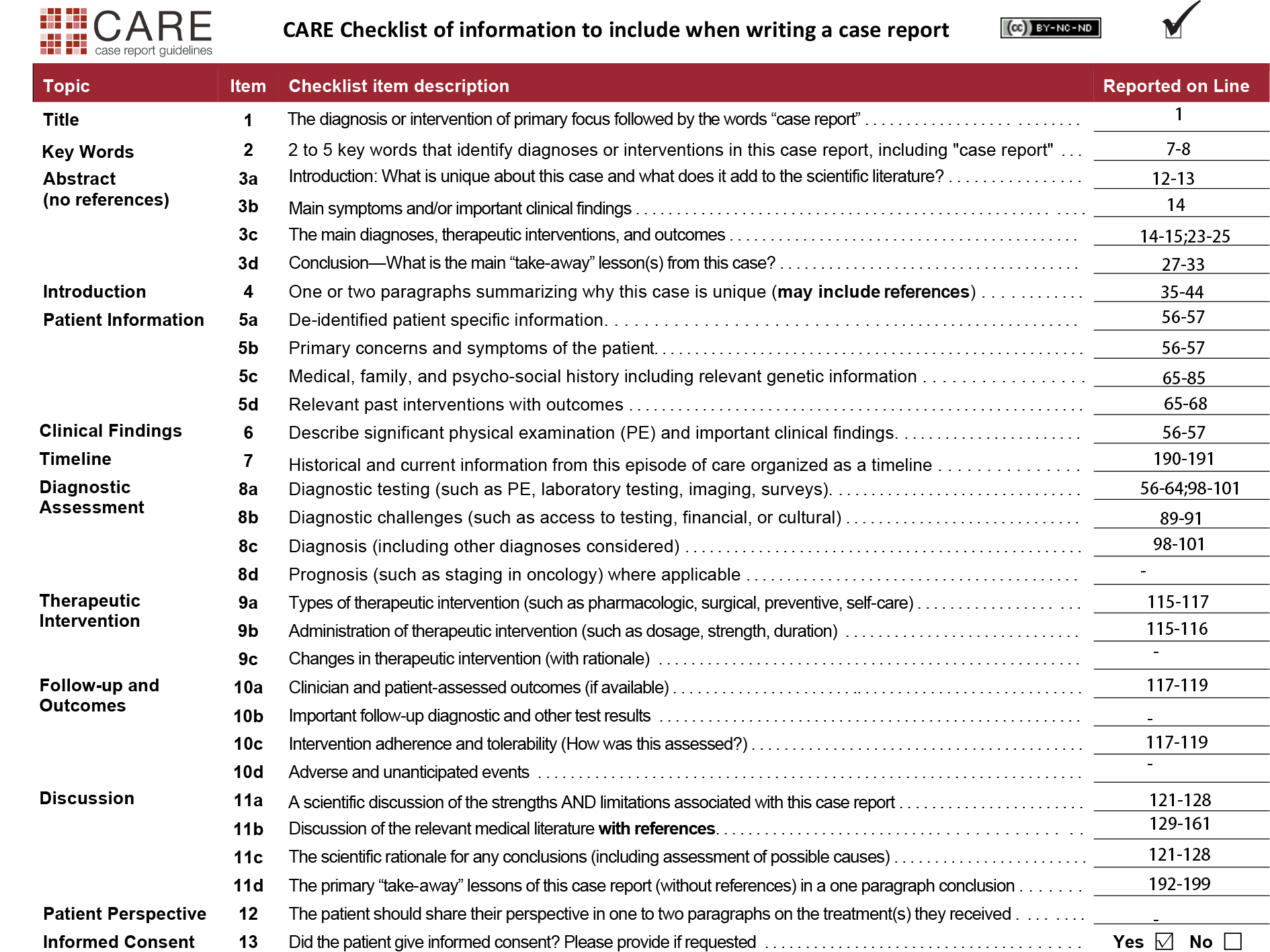

Supplement: Supplementary file 1 [file Image_1.tif]

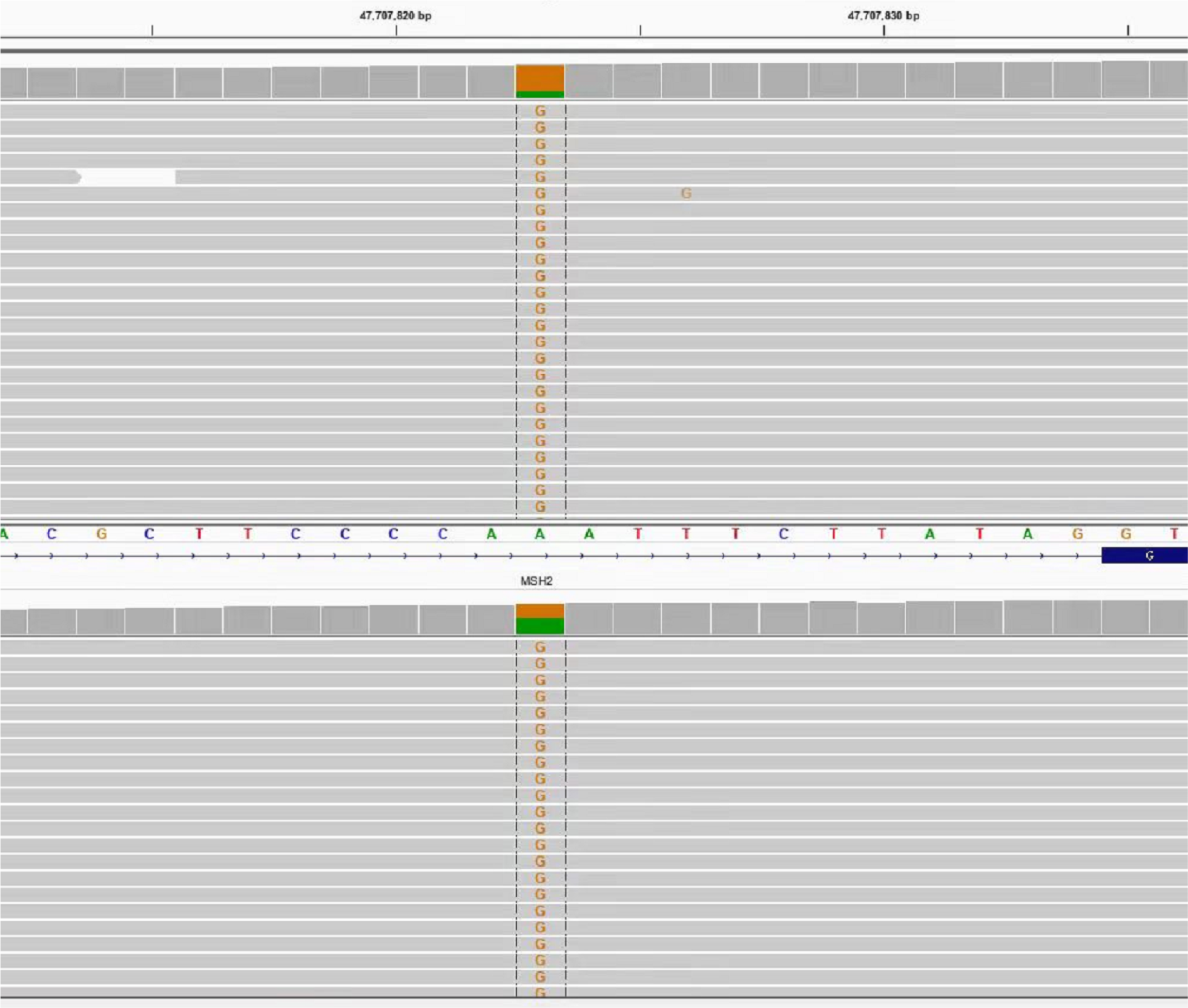

Supplement: Supplementary file 2 [file Image_2.tif]

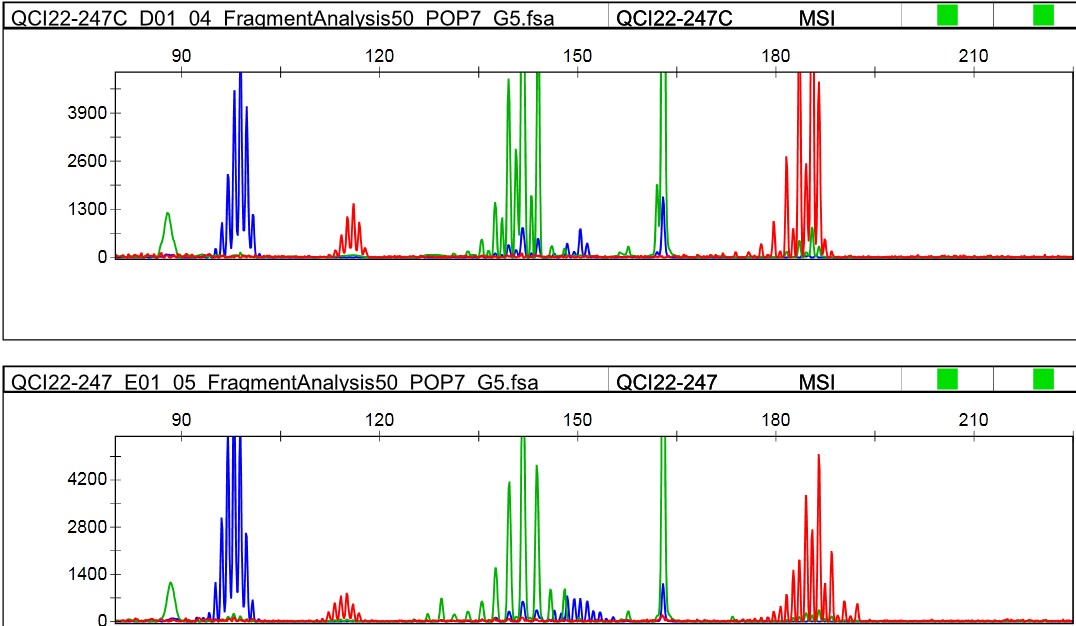

Supplement: Supplementary file 3 [file Image_3.jpeg]
