# Supplementary material for: Case report: Undifferentiated sarcoma with multiple tumors involved in Lynch syndrome: Unexpected favorable outcome to sintilimab combined with chemotherapy
Source: Front Oncol. 2022 Nov 15;12:1014859. doi: 10.3389/fonc.2022.1014859 (PMC9706001; doi:10.3389/fonc.2022.1014859)
Supplement: Supplementary file 4 [file Table_1.docx]

**Supplementary**

**Supplementary Table 1** Summary of results of immunohistochemical analysis, special staining and in situ hybridization of left neck biopsy tissue

| **positive markers** | **negative markers** | |
| --- | --- | --- |
| (+/partially +) | (-) | |
| CyclinD1 | PCK | CK7 |
| SATB2 | CK20 | Villin |
| CD99 | CDX2 | PSAP |
| INI1 | CK8/18 | PAS (special staining) |
| CD56 (partially +) | CD38 | MUM1 |
| CD138 (partially +) | Kappa | Lambda |
| CD68 (partially +) | CD3 | CD20 |
| Ki67 (LI: 40%) | MPO | CD43 |
|  | LCA | CD117 |
|  | ERG | CD34 |
|  | Desmin | S100 |
|  | S0X10 | HMB45 |
|  | MelanA | EBER (in situ hybridization) |
